# Linear FBG Temperature Sensor Interrogation with Fabry-Perot ITU Multi-wavelength Reference

**DOI:** 10.3390/s8106769

**Published:** 2008-10-29

**Authors:** Hyoung-Jun Park, Minho Song

**Affiliations:** Division of Electronics and Information Engineering, Chonbuk National University, Jeonju 561-756, Korea; E-mail: spacegon@chonbuk.ac.kr (H. J. P.)

**Keywords:** Fiber Bragg grating, Fabry-Perot filter, Fiber-optic temperature sensor

## Abstract

The equidistantly spaced multi-passbands of a Fabry-Perot ITU filter are used as an efficient multi-wavelength reference for fiber Bragg grating sensor demodulation. To compensate for the nonlinear wavelength tuning effect in the FBG sensor demodulator, a polynomial fitting algorithm was applied to the temporal peaks of the wavelength-scanned ITU filter. The fitted wavelength values are assigned to the peak locations of the FBG sensor reflections, obtaining constant accuracy, regardless of the wavelength scan range and frequency. A linearity error of about 0.18% against a reference thermocouple thermometer was obtained with the suggested method.

## 1. Introduction

Electric power systems used in the power industry, such as power generators, GIS (gas insulated switchgear), transmission cables, etc., should be operated well below the maximum temperature stipulated by their insulation class specifications, in order to avoid system failures, which can result in immense losses in service quality, as well as in profits. In order to protect these systems from failure through overheating, reliable distributed temperature monitoring is very important. The direct measurement of hotspot temperatures, however, is not possible with conventional sensors, due to the high voltages present during normal operations, which are typically of the order of tens of kV. Furthermore, the use of a harsh installation environment makes it more difficult to implement bulky conventional sensors that have conductive parts. FBG (fiber Bragg grating) sensors appear to be ideally suited for these applications. There are considerable advantages in using FBG sensors, such as the isolation from high line potentials afforded by the dielectric nature of the optical fibers, their potential to take measurements in the presence of high voltages and magnetic noise fields, their wavelength-multiplexing capability, their linear response over a wide temperature range and their remote, high-speed measurement capability, as well as their compactness, light weight and potentially low cost. In principle, hundreds of FBG sensors can be multiplexed with serial and/or parallel sensor array geometry, and there has been a growing need for demodulators that can provide precise, accurate and reproducible determination of the wavelengths reflected by many FBG sensors. Several demodulators have been implemented for this purpose, and the tunable passband filter [1] and CCD spectrometer methods [2] have become the most widely used techniques in this field. The former offers a high signal-to-noise ratio and the better wavelength resolution, while the latter enables a simpler and more robust structure at lower cost.

**Figure 1.**
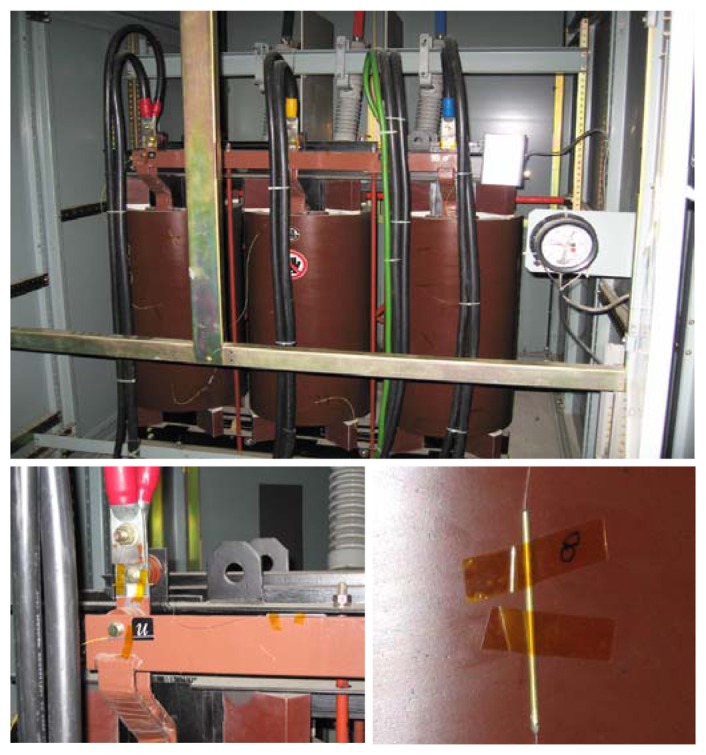
Application of FBG temperature sensors to high-voltage mold type transformers.

In our FBG sensor system, which was applied to the in-use high-voltage mold type transformer shown in figure 1, we used tunable filter demodulation to analyze a sensor array with 15 gratings in the wavelength range of 1539.1∼1561.9 nm.

Compared to our previous system with a lower number of gratings, the linearity of the wavelength scanning became worse due to the wider scan range, resulting in unacceptable measurement errors. Chan *et al.* used gas absorption lines as a multi-wavelength reference to suppress these uncertainties [3], and several fitting algorithms have been proposed to improve the accuracy of peak positioning from FBG profiles [4]∼[8]. In this paper, we used an FPIF (Fabry-Perot ITU filter) as a multi-wavelength reference, and applied a polynomial fitting algorithm to compensate the nonlinear scanning characteristics of the wavelength scanning filter. We obtained a measurement error of 0.18 % with respect to the reference thermocouple thermometer.

## 2. Wavelength interpolation using FPIF spectrum

**Figure2.**
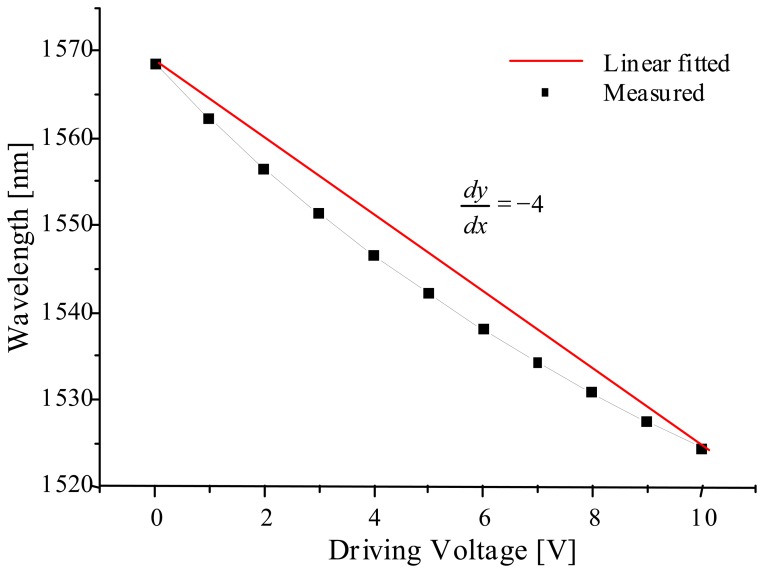
The nonlinear wavelength tuning of the MEMS FP-TF module.

FFP-TFs (fiber Fabry-Perot tunable filters) use high resolution piezoelectric transducers (PZTs) to tune the optical cavity, thereby accomplishing sub-picometer resolution in sensing the Bragg wavelength shifts [4]. Despite their high resolution, FFP-TFs lack long-term stability, because the refractive index of the optical fiber is sensitive to ambient temperature variations, which changes the optical cavity length and passband wavelengths, thus requiring frequent recalibrations. To alleviate this problem, we used an FP-TF fabricated with MEMS (micro-electromechanical systems) technology. The FP-TF (AXSUN) has an FSR (free spectral range) of 80 nm and passbands of 4 GHz width near the center wavelength of the SLD (superluminescent diode) light source (λ_o_∼1550 nm, Δλ∼71.8 nm, P_o_∼ 3 mW ). Because it doesn't have any temperature-sensitive fiber-optic components in its cavity, the passband wavelength stability is maintained at the level of several picometers over a period of several days. However, its wavelength tuning characteristics turned out to be more nonlinear than the FFP-TF, as shown in figure 2, according to the control voltage applied to the driving circuitry.

**Figure3.**
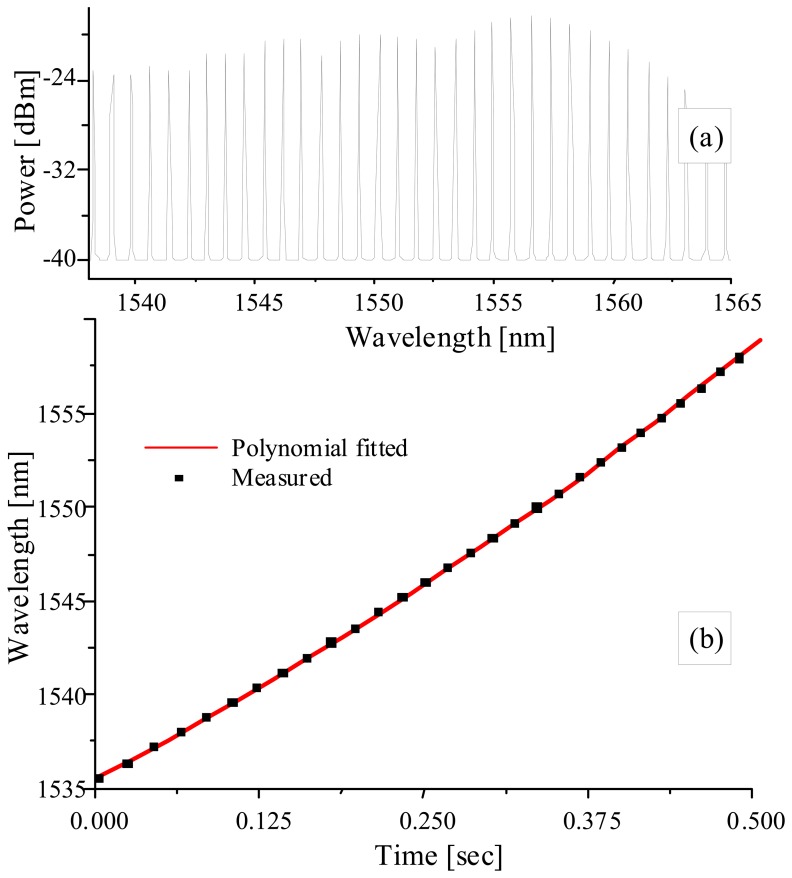
Equidistant multi-wavelengths spectrum of FPIF.

The tunable-filter demodulation technique becomes reliable only when the wavelength tuning is linear or at least repeatable against the ramp driving voltage. The wavelength tuning of the MEMS FP-TF is not only nonlinear, but is also highly dependent on the frequency and the range of the wavelength scan, which makes it difficult to use a look-up table for processing. In previous research with FFP-TFs, we used two athermal-packaged reference gratings (dλ/dT<0.74 pm/°C) that have far-end wavelengths in the FBG array [8, 9]. The wavelength range between the two fixed wavelengths was linear-fitted to minimize the uncertainties caused by random passband variations and quantization errors in the sampling process. However as the scan range and nonlinearity increased, the measurement error with 2 reference gratings increased to an unacceptable level in our specifications. The most straightforward method of solving this problem is to use more fixed reference wavelengths. We found that the commercial FPIF (Fabry-Perot ITU filter) could provide equidistant multi-wavelength references for this purpose. Figure 3(a) shows the multi-passbands of the FPIF with a spacing of 100 GHz and Fig. 3(b) shows the temporal peak distribution scanned by the FP-TF.

With the known wavelength spacing, the temporal wavelength distribution can be obtained by interpolating the peaks.

## 3. Temperature measurements with the FPIF reference

**Figure4.**
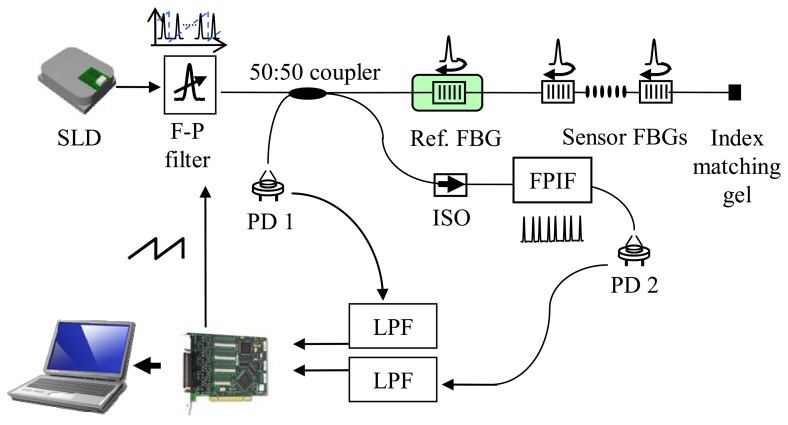
Schematic diagram of the FBG temperature sensor system. (SLD: superluminescent diode, LPF: low pass filter, ISO: isolator, PD: photo diode, FPIF: Fabry-Perot ITU filter).

Figure 4 shows a schematic diagram of the FBG temperature sensor system with the FPIF reference.

**Figure5.**
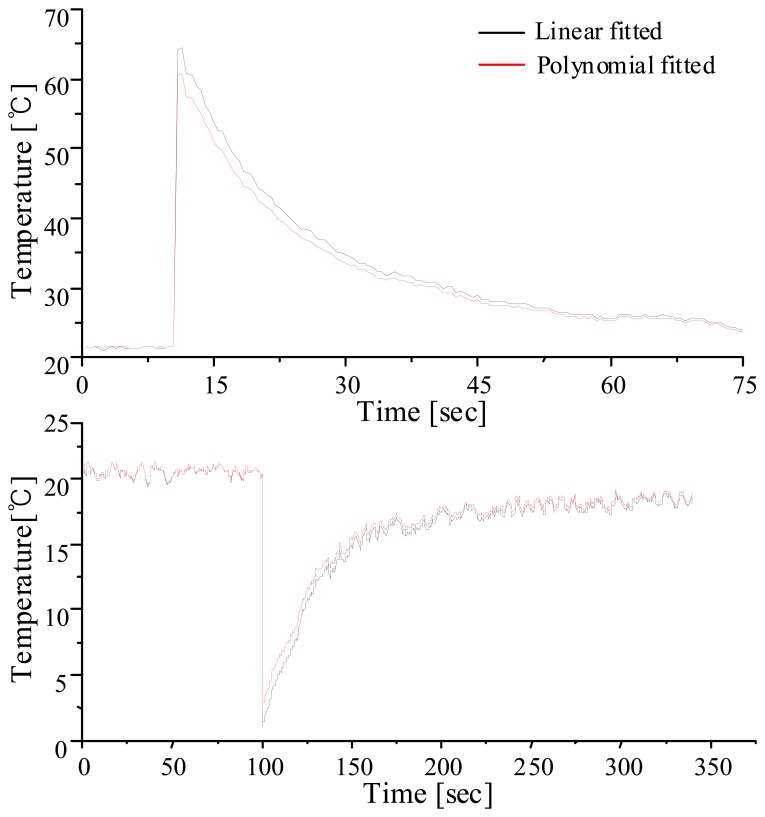
Temperature measurements with linear and polynomial fitting methods.

The light from the SLD passes through the FP-TF that is scanned with a 2 Hz reverse ramp signal, and is directed to the FBG sensor array and FPIF via a 2×2 directional coupler. The light reflected from the FBG array is detected at PD1, and the transmission spectrum of the FPIF is detected at PD2. The lowpass filtered PD outputs are processed in order to locate the peaks in each signal. When the peak locations of the FBG reflections are determined, their wavelengths are given by interpolating the reference wavelengths. A reference grating is temperature-shielding packaged and used to give absolute values in the wavelength calculation. Figure 5 shows the temperature measurement examples; The upper and the lower traces show the temperature variations when a grating sensor was put into a hot and a cold water vessels. The red lines were obtained with the suggested polynomial technique and the black lines were the results of the linear fitting of the previous system. It is clearly shown that the difference between the two schemes increases when the temperature variation becomes faster. The maximum discrepancy was 1.6 °C, which corresponds to ∼9% of the measurement range.

**Figure6.**
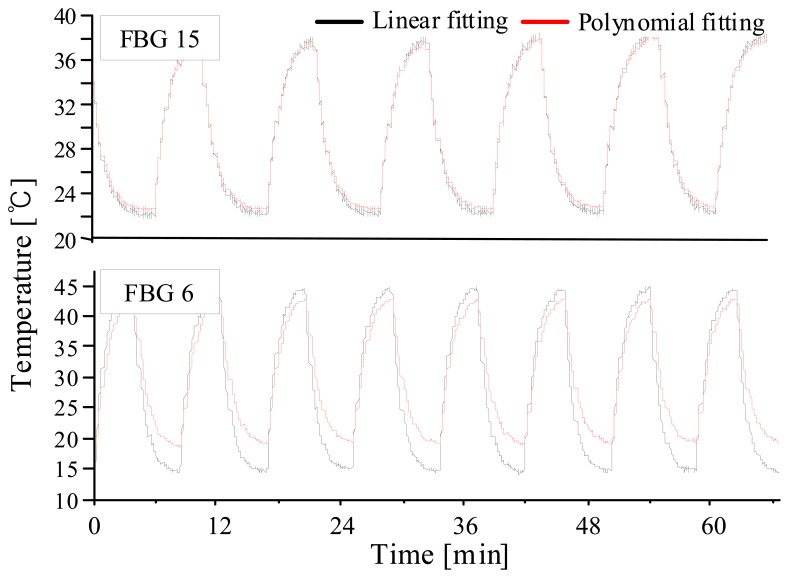
Temperature measurements with a temperature modulator.

**Figure7.**
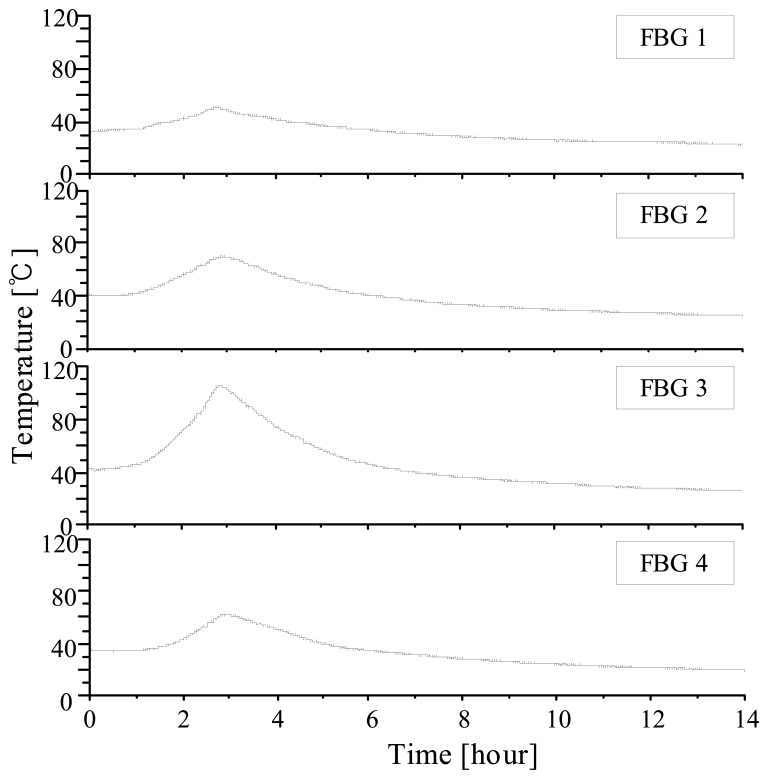
Temperature variations of the high-voltage transformer.

The efficiency of nonlinearity compensation is more clearly shown when we compare two FBG sensors which are located in the middle and at the far end of the scan range. We chose the 6th and 15th gratings that have center wavelengths of 1552.9 and 1561.9 nm, respectively, and attached on thermoelectric cooler and then, applied periodic temperature variations using a temperature modulator. In figure 6, the 15th grating shows little difference between the two schemes, while the 6th grating shows a three times bigger difference between the two processing algorithms. Figure 7 shows 14 hours' temperature variations of 4 locations on a high-voltage transformer. The 3rd grating, which was attached on a bushing of the transformer, showed the highest temperature drifts.

**Figure8.**
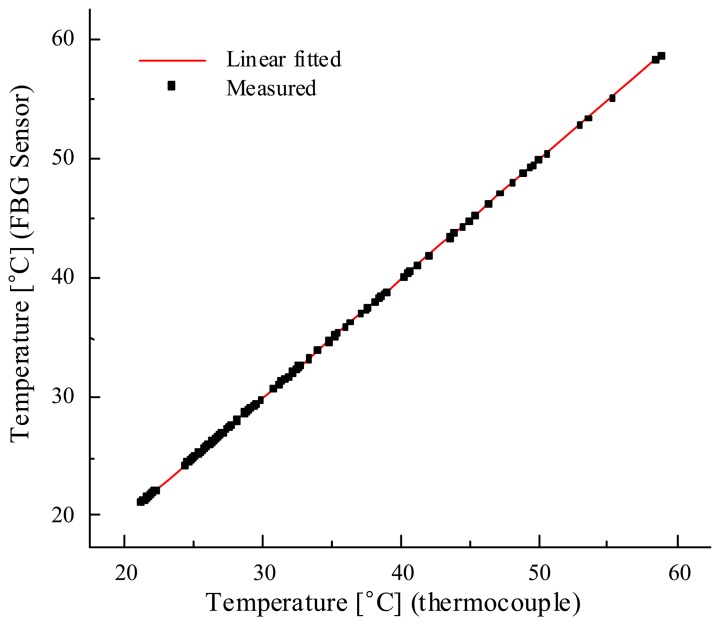
FBG sensor vs. reference thermocouple thermometer.

To verify the accuracy of the proposed technique, we also compared the temperature measurements with a reference thermocouple thermometer and plotted the results in figure 8. In the temperature range of 20 ∼ 60 °C, the linearity error between the two measurements was less than 0.18%.

## 4. Conclusion

We constructed an FBG sensor system with a sensor array of 15 FBGs for the distributed temperature monitoring of electrical power systems. To minimize the effect of nonlinear wavelength scanning, the FBG wavelengths were calculated by interpolating the temporal FPIF peaks. The experimental results showed that the suggested technique reduces the measurement errors, especially when the temperature change rate was high. Compared with the reference thermocouple thermometer, a linearity error of about 0.18% was obtained.
